# Systemic DNA and RNA damage from oxidation after serotonergic treatment of unipolar depression

**DOI:** 10.1038/s41398-022-01969-z

**Published:** 2022-05-16

**Authors:** Anders Jorgensen, Kristin Köhler-Forsberg, Trine Henriksen, Allan Weimann, Ivan Brandslund, Christina Ellervik, Henrik E. Poulsen, Gitte Moos Knudsen, Vibe G. Frokjaer, Martin B. Jorgensen

**Affiliations:** 1 Psychiatric Center Copenhagen, Mental Health Services, Copenhagen, Denmark; 2 Neurobiology Research Unit, Copenhagen University Hospital Rigshospitalet, Copenhagen, Denmark; 3 Institute of Clinical Medicine, University of Copenhagen, Copenhagen, Denmark; 4 Department of Clinical Pharmacology, University Hospital Copenhagen, Bispebjerg and Frederiksberg, Denmark; 5 Department of Clinical Immunology and Biochemistry, Lillebælt Hospital, Vejle, Denmark; 6 Faculty of Health Science, Institute of Regional Health Research, University of Southern Denmark, Odense, Denmark; 7 Harvard Medical School, Boston, USA; 8 Department of Cardiology, Copenhagen University Hospital Hillerød, Copenhagen, Denmark; 9 Department of Endocrinology, Copenhagen University Hospital Hillerød, Copenhagen, Denmark

**Keywords:** Depression, Biomarkers

## Abstract

Previous studies have indicated that antidepressants that inhibit the serotonin transporter reduces oxidative stress. DNA and RNA damage from oxidation is involved in aging and a range of age-related pathophysiological processes. Here, we studied the urinary excretion of markers of DNA and RNA damage from oxidation, 8-oxodG and 8-oxoGuo, respectively, in the NeuroPharm cohort of 100 drug-free patients with unipolar depression and in 856 non-psychiatric community controls. Patients were subsequently treated for 8 weeks with escitalopram in flexible doses of 5–20 mg; seven of these switched to duloxetine by week 4, as allowed by the protocol. At week 8, 82 patients were followed up clinically and with measurements of 8-oxodG/8-oxoGuo. Contextual data were collected in patients, including markers of cortisol excretion and low-grade inflammation. The intervention was associated with a substantial reduction in both 8-oxodG/8-oxoGuo excretion (25% and 10%, respectively). The change was not significantly correlated to measures of clinical improvement. Both markers were strongly and negatively correlated to cortisol, as measured by the area under the curve for the full-day salivary cortisol excretion. Surprisingly, patients had similar levels of 8-oxodG excretion and lower levels of 8-oxoGuo excretion at baseline compared to the controls. We conclude that intervention with serotonin reuptake inhibitors in unipolar depression is associated with a reduction in systemic DNA and RNA damage from oxidation. To our knowledge, this to date the largest intervention study to characterize this phenomenon, and the first to include a marker of RNA oxidation.

## Introduction

Most pharmacological agents with clinical efficacy in major depression modulate neurotransmission mediated by serotonin and other monoamines [1]. However, a plethora of other biological effects of these compounds has been uncovered, including an influence on inflammation [2], bioenergetics [3], and neurotrophics [4]. An area that relates closely to these domains is oxidative stress, where the production of Reactive Oxygen Species (ROS) during cellular respiration exceeds the antioxidant potential of a given biological system, thereby causing downstream modifications of crucial macromolecules such as proteins, lipids, and nucleic acids [5, 6]. Oxidative stress on DNA and RNA is a critical mechanism in aging and in a range of age-related disorders, including—but not restricted to—cancer and neurodegeneration [7, 8], and oxidation of telomeric nucleotides is a key regulator of telomere attrition [9]; a phenomenon which has been observed in depression and other mental disorders [10].

Given the established connections between psychopathology (including affective disorders), aging, and mortality [11, 12], a characterization of antidepressant drug influence on levels of nucleic acid damage from oxidation is clinically relevant. As recently summarized [13], data suggest that SSRIs may have antioxidant effects and lower lipid peroxidation in humans. Experimental studies indicate that this effect could be mediated by a reduction of mitochondrial ROS production [14–16]. To our knowledge, only two intervention studies including 45 [17] and 22 [18] participants have investigated whether treatment with SSRIs affects DNA damage from oxidation. Both studies show that SSRI treatment is associated with a reduction of the DNA oxidation marker 8-oxo-7,8-dihydro-2-deoxyguanosine (8-oxodG). Moreover, a pharmaco-epidemiological study found an association between antidepressant use and lower 8-oxodG levels [19]. To date, no studies have investigated RNA oxidation markers in relation to SSRI exposure.

Here, we examine systemic DNA and RNA damage from oxidative stress, as measured by validated urinary markers, in one hundred initially unmedicated patients with unipolar depression, before and after treatment for 8 weeks with a serotonin reuptake inhibitor drug. The patients were characterized in detail with respect to diagnostic, psychopathological, anthropometric, and biochemical contextual data, allowing for a comprehensive analysis of DNA/RNA marker changes vs. clinical response and a range of other candidate predictor variables, including markers of inflammation and HPA-axis activity [20]. We hypothesized that at baseline patients would have higher levels of systemic DNA and RNA damage from oxidative stress than controls, and that treatment would be associated with a reduction in systemic DNA/RNA damage.

## Patients and methods

### Patients

A detailed study protocol was published previously [20]. One hundred antidepressant drug-free outpatients with moderate to severe unipolar depression were recruited from the mental health system in the capital region of Denmark and included in a non-randomized, 12-week longitudinal, open clinical trial where they received standard antidepressant drug treatment. Patients between 18–65 years of age and with a total score of >17 on the Hamilton Depression Rating Scale 17 items (HAMD_17_) [21] were included. Patients were screened with the Mini International Neuropsychiatric Interview [22] and the diagnosis was confirmed by a specialist in psychiatry. Exclusion criteria were: use of antidepressant medicine within the last 2 months; duration of the present depressive episode exceeding 2 years; more than one attempt with antidepressant treatment in the current episode; previous non-response or known contraindications to an SSRI drug, another primary axis I psychiatric disorder; alcohol/substance abuse or dependence; severe somatic illness; insufficient language skills in Danish; acute suicidal ideation or psychosis; current or planned pregnancy or breastfeeding; use of medical treatment affecting CNS (e.g., metoclopramide, ondansetron, serotonergic drugs for migraine, clonidine); contraindications to PET/MRI scans; history of severe brain injury or significant cognitive impediments. Inclusion took place from August 2016 to March 2019. The primary outcomes of the study with respect to neuropsychological profile [23] and neuroimaging data [24, 25] have been reported elsewhere.

### Study assessments and treatment course

Before inclusion, medical history and prior medical treatment were assessed. All patients underwent somatic and psychiatric screening, urine screening for pregnancy or toxicology, and routine blood tests. After completion of the baseline program, patients started antidepressant treatment with escitalopram, individually adjusted to 10–20 mg daily. Clinical treatment response was monitored after 1, 2, 4, 8, and 12 weeks of treatment by face-to face visits and HAMD_17_ and HAMD_6_ ratings [26]. Regular co-ratings between study investigators were implemented. Patients with intolerable side effects or <25% reduction from baseline in HAMD_6_ at week 4 were offered to switch to the serotonin-norepinephrine reuptake inhibitor, duloxetine, individually adjusted (30–120 mg daily). Compliance was assured by tablet counts and determination of serum escitalopram or duloxetine at week 8. Definitions of remission and response followed the study protocol [20], i.e., remitters were defined as having a ≥50% reduction in HAMD_6_ at week 4 (early responders) and HAMD_6_ score <5 at week 8. Non-responders had <25% reduction in HAMD_6_ at week 4 (early non-responder) and <50% reduction in HAMD_6_ at week 8. Patients in between these categories were referred to as intermediate responders.

### Non-psychiatric community controls

Background population levels of DNA/RNA damage from oxidation were established by data from two large Danish community cohorts with available 8-oxodG/8-oxoGuo levels; The General Suburban Population Study (GESUS) [27, 28], and non-diabetic controls from the Vejle Diabetes Biobank (VDB) [29]. Spot urine samples were obtained from 2007–2010 (VDB) and 2010–2013 (GESUS) and stored below −20 °C before 8-oxodG/8-oxoGuo analysis, which was performed by the same analytical method as in patients (see description below). Potential modifiers of the oxidative stress marker excretion which were also available in the patient population (sex, age, smoking status, body mass index, mean plasma glucose, and plasma creatinine) were retrieved from the cohort databases. On all cohort participants, data on psychiatric diagnoses were retrieved from The Danish Psychiatric Central Research Register (PCRR) [30]. This register contains information on all psychiatric hospital admissions in Denmark since 1969; and from 1995, data on outpatient treatment and emergency room contacts are also included in the register. Psychotropic treatment data were obtained from the Danish national prescription registry, which includes all redeemed prescriptions from any Danish pharmacy from 1995 onwards. Using these data, we excluded individuals with register-based evidence of psychiatric illness, as defined by either (1) a registered World Health Organization (WHO) International Classification of Diseases (ICD-10) diagnosis in the chapters F0-F9 (follow-up period 1995–2018), or (2) more than two redeemed prescriptions of a psychotropic drug (follow-up period Jan 1, 1995–Jun 30, 2017). Due to the age distribution of the patients in the present study, we only included individuals <45 years of age, yielding a total non-psychiatric community control population of 856 individuals. The control population was not matched in sex, and this issue was addressed in a sensitivity analysis (see results). Due to restraints in the extraction of data from the Statistics Denmark database, only aggregated, group-level data were used for comparisons to the patient population.

### Determination of urinary 8-oxodG/8-oxoGuo by ultra-performance liquid chromatography with tandem mass spectrometry

The analyses of the urinary nucleic acid oxidation markers (in both patients and community control samples) were performed with ultra-performance liquid chromatography-tandem mass spectrometry (UPLC-MS/MS) using the Acquity UPLC system and Xevo TQ-S triple quadrupole mass spectrometer purchased from Waters Corp., Milford, USA [31]. UPLC-MS/MS is a validated and highly precise method for measuring urinary nucleic acid oxidation products [32]. The markers do not show diurnal variation [33] and are highly stable for 10+ years when stored at −20 °C [34]; a finding which was reproduced when using the data from the cohorts of the present study (see Supplementary Material). The spot urine sample analyses of 8-oxodG (DNA) and 8-oxoGuo (RNA) were corrected for urinary creatinine levels. The urinary creatinine levels were analyzed by the Jaffe method [35]. The urinary excretion values are expressed as nmol/mmol creatinine.

### Determination of salivary cortisol excretion

Serial saliva samples were sampled at home and collected at baseline and at week 8. Those visits were placed as close to the PET-scan day or week 8 visit as possible, and comprised sampling immediately after awakening and again after 15, 30, 45, and 60 min, at 12, 6, and 11 pm. Participants were instructed to collect saliva samples preferably during weekdays, not perform strenuous exercise <2 h and not to have any oral intake or brush their teeth <1 h prior to sampling. All participants received careful training in saliva collection, instructions on home-sampling procedures; cold storage of samples, and fast delivery either by mail or personal delivery to the laboratory facility for preparation. When received, salivary test tubes were centrifuged and stored at −80 °C until later analysis in one single batch. Salivary cortisol concentrations were determined by a chemiluminescence immunoassay (CLIA) method on the IDS-iSYS automatic analyzer (IDS PLC, Boldon, UK). The intra- and inter-assay variation was <15%, which adhered to the standards of the lab. Based on our previous findings on the relationship between DNA/RNA damage from oxidation and HPA-axis activity [36, 37], we focused the analysis on total cortisol “exposure”, as measured by the area under the curve of the full-day profile of salivary cortisol (AUC_full_), calculated as previously described [38].

### Determination of high-sensitivity c-reactive protein

High-sensitivity c-reactive protein (hsCRP) was determined from serum stored at −20 °C during the project period and analyzed in one batch on a Cobas 8000 with a c502 module by a latex particle-based immunoassay (LIA) turbidimetry method. The lower detection limit was 0.30 mg/L and the upper limit was 20 mg/L. Samples with a CRP concentration below the detection limit were set to 0.30 mg/L. hsCRP measures which were higher than 20 mg/L were subsequently determined as CRP by routine. The coefficient of variation was a maximum 4% for measurements of ~7 mg/L and a maximum 7% for lower measures of ~0.6 mg/L.

### Statistics

The study was preceded by a power analysis. Hence, using previously obtained means and standard deviations of 8-oxodG in control populations (1.4 (0.3) nmol/mmol creatinine), the inclusion of 96 individuals in each group would yield 90% statistical power to detect a 10% difference between patients and controls at an alpha-level of 0.05. The primary analyses of the study, pertaining to the primary hypotheses, were baseline comparisons of 8-oxodG/8-oxoGuo excretion levels in patients and controls and the pre-post change in 8-oxodG/8-oxoGuo excretion in patients. All other analyses were considered secondary and exploratory. Baseline levels of 8-oxodG/8-oxoGuo in patients and community controls were compared with independent samples *t*-tests using means, standard deviations, and sample size of the patient and control population, respectively. Pre-post treatment changes in 8-oxodG/8-oxoGuo were compared with paired samples *t*-tests. The predictive value of the 8-oxodG/8-oxoGuo markers was analyzed by comparing baseline marker excretion to week 8 response status with analysis of variance (ANOVA). The association between 8-oxodG/8-oxoGuo marker excretion and clinical (week 8 response status, HAM-D scores, BMI) and biochemical variables (AUC_full_, hsCRP, p-escitalopram) were analyzed by Pearson correlation coefficients (continuous variables) or ANOVA (categorical variables). We controlled for baseline differences by using pre-post delta values of the continuous markers. Finally, we used multivariate regression models with backward elimination to estimate the most influential of a range of potential modifiers (age, sex, smoking status, ΔHAMD_6_, ΔBMI, ΔAUC_full_, ΔhsCRP, and post-treatment plasma escitalopram concentration) on 8-oxodG/8-oxoGuo excretion change. If not otherwise stated, data are presented as means (standard deviation), absolute numbers (percentage), or estimates/regression coefficients [95% confidence intervals]. All statistical tests were two-sided. All data preparation and statistical analyses were performed in R Studio (version 1.1.447) with packages “data.table” and “Publish” installed.

### Ethics

The study protocol was approved by all relevant authorities (the Health Research Ethics Committee of the Capital Region of Denmark (H-15017713), the Danish Data Protection Agency (04711/RH-2016-163) and Danish Medicines Agency (EudraCT- 2016-001626-34)). Likewise, the control population cohorts were approved by local ethics authorities (GESUS: SJ-113, SJ-114, SJ-147, SJ-278; VDB: S-20080097), and reported to the Danish Data Protection Agency. All participants provided written and informed consent prior to inclusion.

## Results

Baseline characteristics, changes in depression scores, and week 8 plasma concentrations of the antidepressants of patients and the background population controls are presented in Table 1. Two individuals with non-detectable levels of escitalopram in plasma at week 8 were included in the baseline analysis only. A total of 82 individuals completed the intervention and participated in the follow-up investigations at week 8. Since only seven patients switched to duloxetine, we did not make separate analyses for this subset of patients. At baseline, the mean age of the patients was 27.0 (8.1) years, and 73% were female. The antidepressant intervention was associated with a significant reduction in HAM-D_6_ and HAM-D_17_ scores (*p* < 0.0001 for both scales). Seventeen percent were classified as non-responders; 54% as intermediate responders and 28% as remitters.

**Table 1:** Sociodemographic, clinical, and treatment response data from background population controls, as well as patients with MDD at baseline and week 8 follow-up.

Variable	Controls, full (*N* = 856)	Controls, matched (*N* = 67)	Baseline (*n* = 100)	Week 8 (*n* = 82)
Sex (F (%) / M (%))	511 (59.7) / 345 (40.3)	50 (74.6) / 17 (25.4)	73 (73.0) / 27 (27.0)*	
Age (years)	38.4 (4.6)	28.5 (2.4)	27.0 (8.1)*	
Smokers (*N* (%))	131 (15%)	9 (13.4)	19 (19%)	
BMI (kg/m^2^)	24.5 (4.7)	22.9 (4.4)	24.6 (5.6)^**#**^	
Mean glucose (mmol/L)	5.8 (0.5)	5.7 (0.4)	5.6 (0.9)*	
Plasma creatinine (µM)	74.1 (14.5)	74.8 (16.3)	71.8 (13.2)	
Plasma escitalopram (nM) (*n* = 71)				78.3 (45.3)
Plasma duloxetine (nM) (*n* = 7)				139.6 (82.0)
HAM-D_6_			12.3 (1.6)	5.9 (3.8)**
HAM-D_17_			22.9 (3.4)	11.5 (6.6)**
Treatment response (*N* (%))				
Non-responders				14 (17.3)
Intermediate responders				44 (54.3)
Remitters				23 (28.4)

Signifcant differences between the groups are marked with * (full control population) and ^**#**^ (fully age- and sex-matched control population) vs. patients at baseline, or ** (patients at baseline vs. week 8). Data were analyzed with independent samples *t*-tests, paired samples *t*-tests or chi-squared tests, as appropriate. Data are presented as means (standard deviation) if not otherwise stated.

**Fig. 1:**
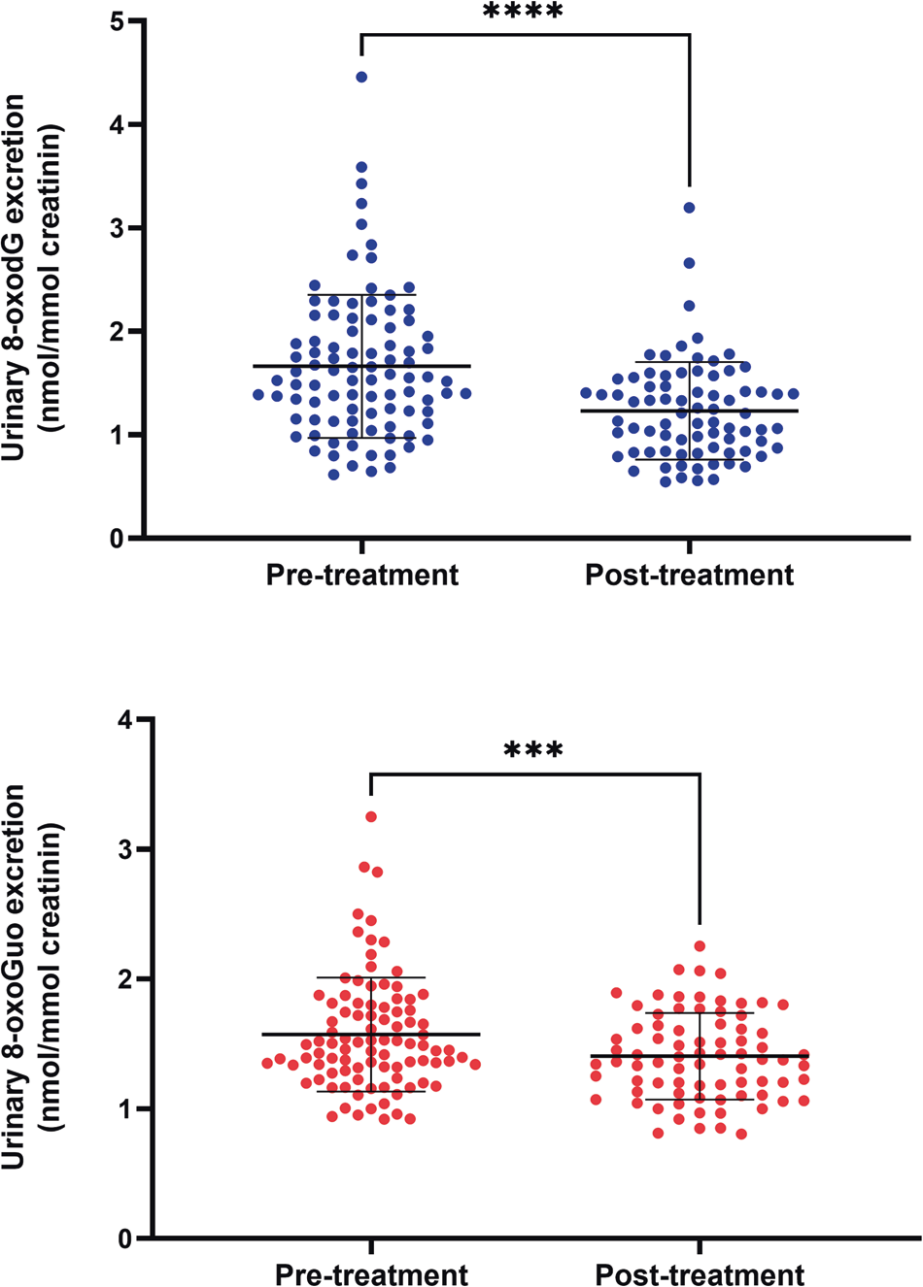
Pre- and post-treatment urinary excretion of markers of DNA and RNA damage from oxidation, 8-oxodG (blue) and 8-oxoGuo (red), respectively, in one hundred antidepressant drug-free patients with unipolar depression treated with a serotonergic antidepressant (escitalopram/duloxetine) for 8 weeks. Data are showed as individual data points, means, and standard deviations, and were analyzed with paired *t*-tests. ****p < 0.0001, ***p < 0.001.

We found a highly significant reduction in the excretion of both nucleic acid oxidation markers at week 8 compared to baseline (8-oxodG: −0.43 [95% CI −0.55 to −0.32] nmol/mmol, t = −7.70, *p* < 0.0001. 8-oxoGuo: −0.16 [95% CI −0.24 to −0.07] nmol/mmol, t = −3.68, *p* = 0.0004) (Fig. 1). At baseline, urinary 8-oxodG excretion did not significantly differ between patients (1.66 (0.7) nmol/mmol) and controls (1.63 (0.7) nmol/mmol) (t = 0.39, *p* = 0.70). In contrast, patients had significantly lower levels of 8-oxoGuo (1.57 (0.4) nmol/mmol) compared to controls (1.89 (0.6) nmol/mmol) (t = −6.98, *p* < 0.001). Because the control population did not entirely match the patient population with respect to age (38.4 (4.6) vs. 27.0 (8.1) years) and sex (59% vs 73% female), we did a sensitivity analysis with a better matched—but significantly smaller—control group (*n* = 67, age 28.5 (2.4) years, 75% female) (Table 1), which did not alter the result of similar 8-oxodG and lower 8-oxoGuo levels in patients vs. controls (Table 2). At a Bonferroni-corrected *p*-value of 0.025, these results continued to be significant. In patients, baseline symptom severity as measured by HAM-D_6_ or HAM-D_17_ was not correlated to baseline levels of either marker (HAM-D_6_, 8-oxodG: r = −0.10 [−0.29 to 0.11], *p* = 0.36; HAM-D_17_, 8-oxodG: r = −0.06 [−0.26 to 0.14], *p* = 0.56; HAM-D_6_, 8-oxoGuo: r = 0.12 [−0.32 to 0.08], *p* = 0.23, HAM-D_17_, 8-oxoGuo: r = 0.09 [−0.28 to 0.12], *p* = 0.40).

**Table 2:** Urinary excretion of markers of DNA and RNA damage from oxidation, 8-oxodG and 8-oxoGuo (nmol/mmol creatinine), respectively, in the full control population, the age- and sex-matched control population, and in one hundred antidepressant drug-free patients with unipolar depression pre- and post-treatment with a serotonergic antidepressant (escitalopram/duloxetine) for 8 weeks.

	Controls, full (*N* = 856)	Controls, matched (*N* = 67)	Patients (baseline) (*N* = 100)	Patients (week 8) (*N* = 82)
8-oxodG	1.6 (0.7)	1.6 (0.8)	1.7 (0.7)	1.2 (0.5)*^,^**
8-oxoGuo	1.9 (0.6)	1.8 (0.5)	1.6 (0.4)*	1.4 (0.3)*^,^**

Data are showed as means and standard deviations and were analyzed with independent or paired *t*-tests, as appropriate.

*Significantly different from both control groups (*p* < 0.001).

**Significantly different from pre-treatment (*p* < 0.001).

**Fig. 2:**
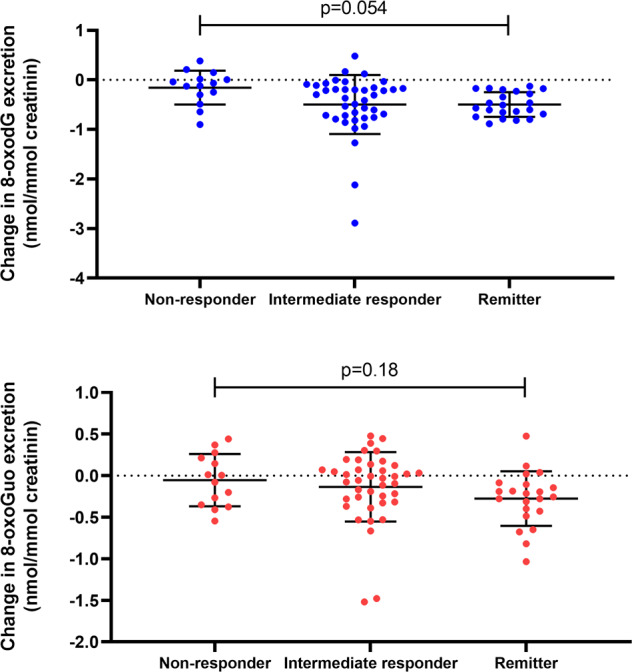
The relationship between the urinary excretion of markers of DNA and RNA damage from oxidation, 8-oxodG (blue) and 8-oxoGuo (red), respectively, in patients with unipolar depression, as compared to response status after 8 weeks of treatment with a serotonergic antidepressant (escitalopram/duloxetine). Data are showed as individual data points, means, and standard deviations, and were analyzed with ANOVA.

The relation between clinical response and change in 8-oxodG/8-oxoGuo excretion is shown in Fig. 2. We found a borderline significant trend towards an association between the 8-oxodG pre-post intervention change and categorical response status (F(2,73) = 2.91, *p* = 0.054), with all remitters showing a reduction in 8-oxodG excretion as compared to the non-responders and intermediate responders. The association between 8-oxoGuo excretion change and response status was not significant (Fig. 2). Baseline 8-oxodG/8-oxoGuo excretion did not predict drug treatment response at week 8 (8-oxodG: F(2,77) = 0.52, *p* = 0.59; 8-oxoGuo: F(2,77) = 0.37, *p* = 0.69).

**Fig. 3:**
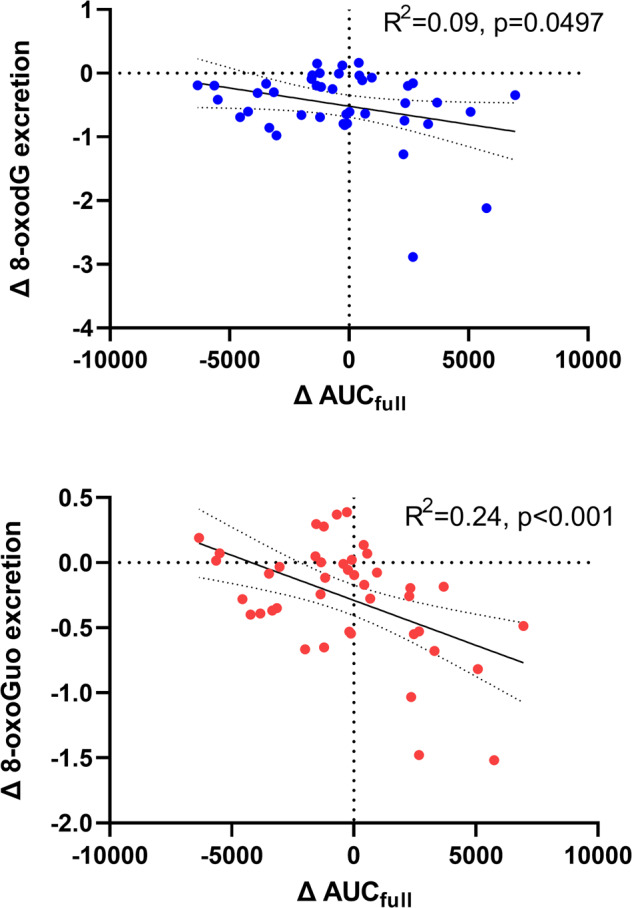
Correlations between the pre-post treatment change in urinary excretion of markers of DNA and RNA damage from oxidation, 8-oxodG (blue) and 8-oxoGuo (red), respectively, vs. pre-post treatment change in area under the curve for the full-day profile of salivary cortisol excretion (AUC_full_). Data are showed as individual data points, regression lines with 95% confidence intervals, and were analyzed by linear regression.

We explored the associations between a range of hypothesized determinants of oxidative stress and 8-oxodG/8-oxoGuo changes (HAM-D scores, BMI, full-day cortisol output (AUC_full_), hsCRP, and plasma escitalopram). The only significant finding was a negative association between the change in AUC_full_ and the change in 8-oxodG and 8-oxoGuo excretion (8-oxodG: r = −0.30 [−0.55 to −0.001], *p* = 0.0497. 8-oxoGuo: r = −0.49 [−0.69 to −0.22], *p* = 0.0009) (Table 3 and Fig. 3). Note, however, that the association to change in 8-oxodG was only borderline significant and no longer significant (*p* = 0.08) after the removal of the outlier. Correspondingly, in multivariate regression analyses with backwards elimination, the only variable surviving the sequential removal of non-significant variables, was AUC_full_, both with respect to 8-oxodG (*N* = 44, *p* = 0.049) and 8-oxoGuo (*N* = 44, *p* = 0.0009). Due to missing samples, it was only possible to calculate the pre-post intervention change in AUC_full_ in 44 patients.

**Table 3:** Pearson correlation estimates and *p*-values for the association between pre- to post-treatment change in 8-oxodG (Δ8-oxodG) and 8-oxoGuo (Δ8-oxoGuo), respectively, vs. pre- to post-treatment change in the predictor variables depressive symptoms (ΔHAM-D_6,_ ΔHAM-D_17_), body mass index (ΔBMI), area under the curve of salivary cortisol excretion (full-day profile) (ΔAUC_full_), high-sensitivity c-reactive protein (ΔhsCRP), and post-treatment plasma escitalopram concentration.

	ΔHAM-D_6_	ΔHAM-D_17_	ΔBMI	ΔAUC_full_	ΔhsCRP	p-escitalopram
Δ 8-oxodG	0.15 *p* = 0.21	0.15 *p* = 0.18	−0.14 *p* = 0.21	**−0.30** ***p*** = **0.0497**	−0.04 *p* = 0.74	0.006 *p* = 0.96
Δ 8-oxoGuo	0.15 *p* = 0.20	0.15 *p* = 0.21	−0.03 *p* = 0.77	**−0.49** ***p*** = **0.0009**	−0.16 *P* = 0.16	0.24 *p* = 0.051

Significant associations are in bold.

## Discussion

In the present study, we tested the hypothesis that treatment with serotonergic antidepressants would be associated with a reduction in systemic nucleic acid damage from oxidation in a large sample of initially drug-free patients with unipolar depression. To our knowledge, this is to date the largest clinical intervention study investigating the effects of antidepressant treatment on levels of systemic DNA damage from oxidation, and the first to investigate levels of RNA damage from oxidation in this context. We found a highly significant reduction in both the DNA and the RNA oxidation marker after treatment. We have previously found that urinary 8-oxodG and 8-oxoGuo levels are higher in people with severe psychiatric disorders as compared to healthy controls [39–41]. We have also shown that 8-oxoGuo, but not 8-oxodG, is increased in patients hospitalized with severe depression and treated with antidepressant drugs. Surprisingly, 8-oxoGuo was further increased after electroconvulsive therapy (ECT) in these patients [40]. The fact that two clinically efficacious treatments of depression with highly different modes of action, i.e., ECT and SSRIs, can have opposite effects on the same nucleic acid oxidation marker suggests that the phenomenon is not causally related to the antidepressant effect. This is supported by our present finding that the marker reductions were not significantly correlated to clinical response, although there was a trend towards a reduction of 8-oxodG in the responders compared to the intermediate and non-responder groups.

What mechanism could underlie a reduction of nucleic acid oxidation by a serotonergic antidepressant drug? Fanibunda et al. recently demonstrated that in cortical neurons, serotonin enhances mitochondrial respiration; reduces ROS production, and increases the expression of antioxidant enzymes superoxide dismutase 2 and catalase. These effects were mediated by the 5-HT_2A_ receptor and subsequent activation of a mitochondrial modulator pathway, the SIRT1-PCG-1α axis [42]. One natural downstream consequence of such a role of serotonin biology, i.e., reduced ROS and increased antioxidant defenses, would be reduced oxidative modifications of nucleic acids and other macromolecules vulnerable to oxidative stress. Both the serotonin transporter (SERT) and the 5-HT_2A_ receptor are widely distributed outside the CNS, including in platelets and the liver [43]. Based on these observations, we speculate that a plausible explanation for our present finding is that the CNS and/or peripheral blockade of SERT caused by an SSRI, and the resulting increased extracellular 5-HT concentration, could cause reduced intracellular oxidative stress by altering mitochondrial function through 5-HT_2A_ activation.

The outcome markers of the study, 8-oxodG and 8-oxoGuo, were determined by highly sensitive liquid chromatography coupled with tandem mass spectroscopy, which is superior to other methods, e.g., ELISA, in precision and linearity [32]. The method allows for the simultaneous determination of the ribonucleoside and deoxyribonucleoside form of the oxidized guanine nucleoside, representing systemic RNA and DNA oxidation, respectively. The exact subcellular origins of these molecules are not clear, but the cellular release of 8-oxodG is thought to stem from enzymatic repair of DNA and/or the nucleotide pool, whereas the release of 8-oxoGuo is thought to stem from the degradation of RNA, as no known RNA repair mechanism exists. Because the capacity to repair/degrade the oxidized nucleotides by far exceeds the rates by which they are formed, the urinary amounts excreted in steady-state is thought to depend solely on the rate of formation and not on the rate of repair/degradation [5]. We have recently tested and verified this rationale in an in silico model of 8-oxodG and 8-oxoGuo metabolism [44]. Because the organismal origins of the markers cannot be determined from the urinary levels, the markers should be interpreted as indicators of the systemic oxidative stress on DNA/RNA.

Even if a reduction of nucleic acid oxidation is not causally involved in the resolution of depression with treatment, the phenomenon may have other important clinical implications. Oxidative modifications of DNA may cause mutations, cell-cycle arrest, and apoptosis [7]. Furthermore, as mentioned above, oxidation of telomeric DNA regulates the telomere length and thereby influences cellular aging [9]. RNA damage from oxidation has come more into attention within the recent decade, in particular within the fields of neurodegeneration [8]. All RNA species are susceptible to damage from oxidation, including both coding and non-coding RNA subtypes. Oxidative modifications of mRNA may cause truncated proteins, ribosomal stalling, and reduced protein expression levels. Oxidative modifications of non-coding RNAs may cause misrecognition of the target mRNA, which can lead to reduced expression of the associated protein or binding to other mRNAs than the native target [8]. Our group has found that 8-oxoGuo levels are a predictor of mortality in patients with type-2 diabetes and may be mechanistically involved in the development of this disease [45, 46]. Hence, a reduction of DNA and RNA damage from oxidation by SSRIs could be speculated to positively influence the trajectories of increased brain and bodily aging associated with affective disorders [12, 47].

We found that patients had similar levels of 8-oxodG as the background population controls, and—surprisingly—lower levels of 8-oxoGuo. This is in partial contrast with recent findings by Ceylan et al., who found that urinary 8-oxodG was higher in patients with acute unipolar and bipolar depression (42% bipolar, 74% medicated) than in healthy controls. In line with our findings, the remission of symptoms was associated with a reduction of 8-oxodG excretion in a naturalistic follow-up [48]. Apart from their depressive episode, the patients participating in our study had a relatively healthy lifestyle, were screened meticulously for any chronic disorders or drug/alcohol abuse, and were drug-free, whereas the background population controls were only included by their non-psychiatric morbidity status. Hence, we speculate that the finding is explained by the patients having—on average—less metabolic stress than the background population. The finding is in line with the notion that the systemic nucleic acid damage from oxidation is not causally related to depression per se.

We did an exploratory analysis of the association between a range of clinical, anthropometric, and metabolic possible mediators of changes in oxidative stress on DNA and RNA, respectively, including age, sex, week eight plasma escitalopram concentration, as well as changes in BMI, HPA-axis activity and inflammation. Among these, the only variable showing significant associations to changes in DNA/RNA damage from oxidation was AUC_full_, which can be considered a marker of individual overall cortisol “exposure” during the day. This finding adds support to the body of literature suggesting an important biological connection between corticosteroids and oxidative stress [49], which in turn has been suggested to underlie the association between stress, depression, and aging [50]. However, in the present study, the pre- versus post-treatment change in AUC_full_ was negatively associated with 8-oxodG and 8-oxoGuo. The association of AUC_full_ was considerably weaker to 8-oxodG than to 8-oxoGuo. We have previously found positive associations between 24 h urinary cortisol and 8-oxodG/8-oxoGuo excretion [36], but in a more recent experimental study of rats who were administered corticosterone—to reach systemic levels corresponding to those found in chronic psychological stress—we found that the intervention caused significantly reduced levels of 24 h urinary excretion of 8-oxodG, and a similar trend for 8-oxoGuo, as compared to the vehicle-treated animals [37]. Collectively, these findings contradict the notion of a simple positive correlation between corticosteroid exposure and oxidative stress. Possible explanations for these ambiguities include the corticosteroid-induced change in the subcellular sensitivity to corticosteroids due to downregulation of its receptors; indirect antioxidant effects of higher corticosterone (such as less calorie intake); or differential effects on nucleic acid and non-nucleic acid markers of oxidative stress [37]. Future studies are needed to address this question.

The major advantages of the present study include that we used a large and very well-characterized sample of initially drug-free individuals with unipolar depression; that we used a highly validated and precise method for detecting 8-oxodG and 8-oxoGuo; that we—to our knowledge for the first time—studied an RNA oxidation marker in relation to SSRI exposure, and that the study included detailed contextual information on known potential mediators of oxidative stress on DNA/RNA. The primary limitation of the study is that it did not include a placebo group, and although the finding corresponds well with the existing literature, we thus cannot conclude whether the observed reduction in markers of DNA/RNA damage from oxidation after SSRI exposure is causal or whether it represents an antidepressant mechanism of SSRIs. While the week 8 drop-out was within the expected range, some of the markers (in particular AUC_full_) had missing values, leading to reduced statistical strength. The community control group was not completely matched to the patient group with respect to age and sex, but we did sensitivity analyses to account for these discrepancies without changes to the overall findings. Comparable data on other potential confounders such as diet, dietary supplements, exercise levels, etc., was not available across the cohorts. However, we and others have investigated the role of these potential confounders in a range of studies and found their influence on 8-oxodG/8-oxoGuo excretion to be minimal or absent [51–53]. Finally, the control cohort was historical and not recruited from the same catchment area as the patients, and we cannot rule out that this may introduce an unknown bias with respect to 8-oxodG/8-oxoGuo excretion levels. However, we find that the inclusion of a community control population—rather than a “super healthy” control group free of any disease or risk states—has benefits in determining to what extent the baseline findings in patients explain the differences in morbidity and mortality of psychiatric patients found in register-based studies, where the comparison group is usually the background population.

We conclude that, in a very well-characterized population of unmedicated patients with unipolar depression, an intervention with a serotonergic antidepressant was associated with a substantial reduction in systemic markers of DNA and RNA damage from oxidation. The changes were not correlated with overall symptom reduction but showed a trend towards an association to remission status. Future studies must elucidate if serotonergic antidepressant mechanisms include reduction in systemic markers of DNA and RNA damage from oxidation, and to what extent the phenomenon is relevant for longer-term outcomes, such as aging and its related morbidity, in patients with mental disorders treated with SSRIs.

## Supplementary information

Supplementary Material
