# Supplementary material for: Systemic DNA and RNA damage from oxidation after serotonergic treatment of unipolar depression
Source: Transl Psychiatry. 2022 May 16;12:204. doi: 10.1038/s41398-022-01969-z (PMC9110351; doi:10.1038/s41398-022-01969-z)
Supplement: Supplementary file 1 — Supplementary Material [file 41398_2022_1969_MOESM1_ESM.pdf]

Supplementary Material

Systemic DNA and RNA damage from oxidation after serotonergic treatment of unipolar depression.

Jorgensen, A. et al, 2022.

Supplementary figures 1-6: Days in freezer (time from sampling date to time of analysis) in the GESUS (n=535) (**1+2**), Vejle Diabetes Biobank (n=321) (**3+4**), and Neuropharm 1 (n=100) (**5+6**) cohorts vs. creatinine corrected 8-oxodG and 8-oxoGuo marker levels, respectively. Below each plot, the regression coefficient estimates for the freezertime vs. marker level and corresponding p-values are given. The removal of outliers did not change the results.

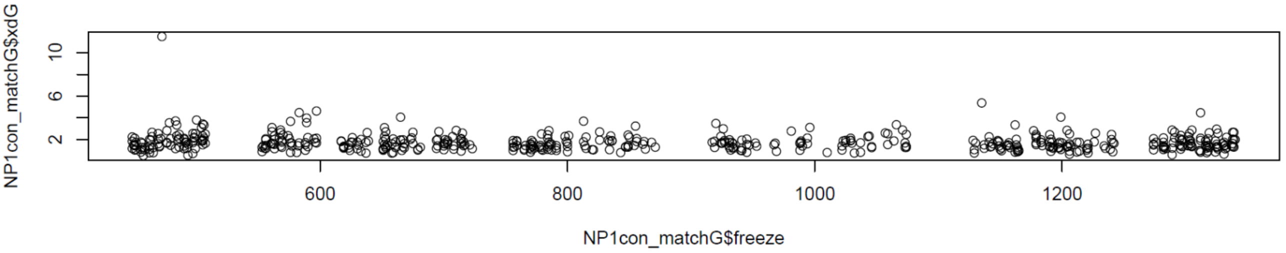

1. Coefficient estimate = -0.000295, p=0.0103

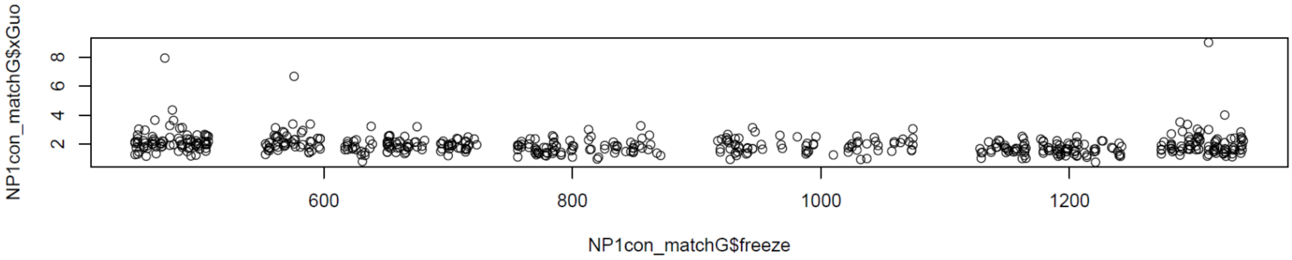

2. Coefficient estimate = -3.335e-04, P=0.000629

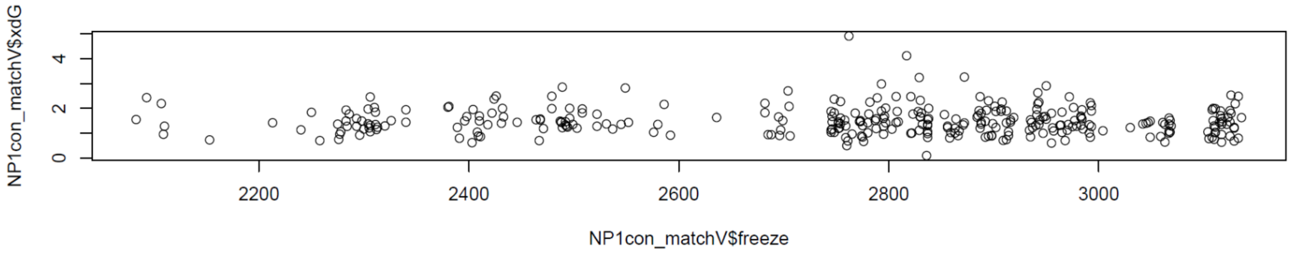

3. Coefficient estimate = -7.105e-05, p=0.541

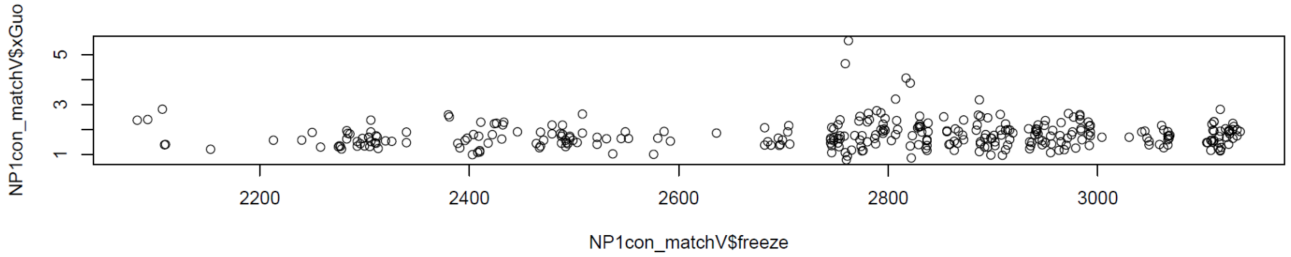

4. Coefficient estimate =0.0001781, p=0.103

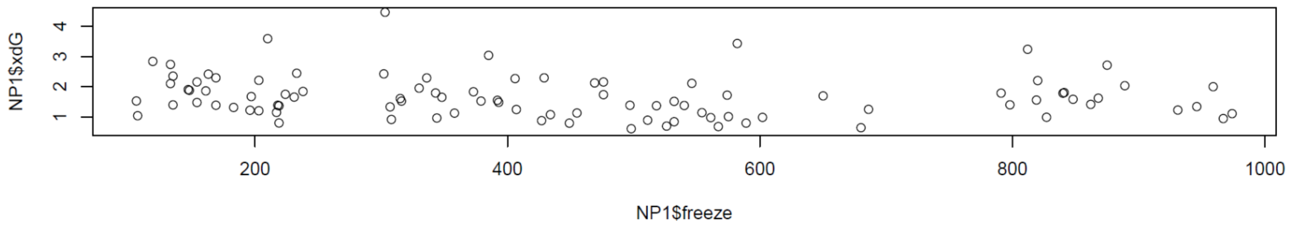

5. Coefficient estimate =-0.0004257, p=0.132

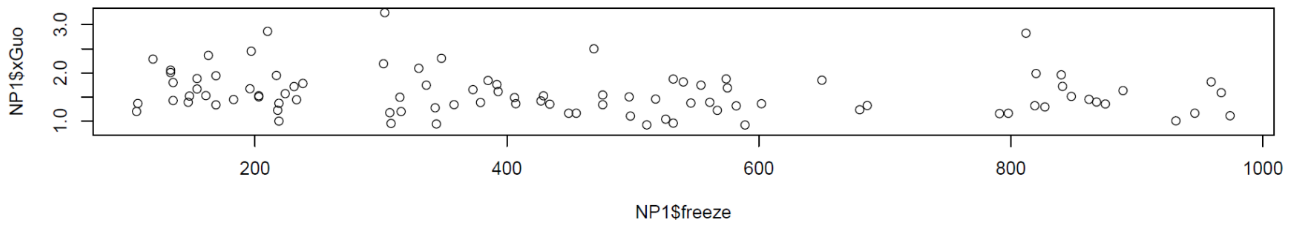

6. Coefficient estimate =-0.0003366, p=0.0602
